# Sugar phosphate-mediated inhibition of peptidoglycan precursor synthesis

**DOI:** 10.1128/mbio.01729-25

**Published:** 2025-07-28

**Authors:** Megan R. Keller, Vijay Soni, Megan Brown, Kelly M. Rosch, Anas Saleh, Kyu Rhee, Tobias Dörr

**Affiliations:** 1 Weill Institute for Cell and Molecular Biology, Cornell University 5922, Ithaca, New York, USA; 2 Cornell Institute of Host-Microbe Interactions and Disease, Cornell University, Ithaca, New York, USA; 3 Department of Medicine, Division of Infectious Diseases, Weill Cornell Medicine 12295, New York, New York, USA; 4 Department of Microbiology, Cornell University 5922, Ithaca, New York, USA; NYU Langone Health, New York, USA

**Keywords:** gram-negative bacteria, bacterial metabolism, antibiotic resistance, stress, enzyme inhibitor

## Abstract

Antibiotic tolerance, the widespread ability of diverse pathogenic bacteria to sustain viability in the presence of typically bactericidal antibiotics for extended time periods, is an understudied stepping stone toward antibiotic resistance. The gram-negative pathogen *Vibrio cholerae*, the causative agent of cholera, is highly tolerant to β-lactam antibiotics. We previously found that the disruption of glycolysis, via deletion of *pgi* (*vc0374*, glucose-6-phosphate isomerase), resulted in significant cell wall damage and increased sensitivity toward β-lactam antibiotics. Here, we uncover the mechanism of this resulting damage. We find that glucose causes growth inhibition, partial lysis, and a damaged cell envelope in ∆*pgi*. Supplementation with N-acetylglucosamine, but not other carbon sources (either from upper glycolysis, TCA cycle intermediates, or cell wall precursors) restored growth, re-established wild-type β-lactam resistance, and recovered cellular morphology of a *pgi* mutant exposed to glucose. Targeted metabolomics revealed the cell wall precursor synthetase enzyme GlmU (*vc2762*, coding for the bifunctional enzyme that converts glucosamine-1P to UDP-GlcNAc) as a critical bottleneck and mediator of glucose toxicity in ∆*pgi*. *In vitro* assays of GlmU revealed that sugar phosphates (primarily glucose-1-phosphate) inhibit the acetyltransferase activity of GlmU (likely competitively), resulting in compromised peptidoglycan and lipopolysaccharide biosynthesis. These findings identify the molecular mechanism of Δ*pgi* glucose toxicity in *V. cholerae*.

**IMPORTANCE:**

Sugar-phosphate toxicity is a well-characterized phenomenon that is seen within diverse bacterial species, and yet the molecular underpinnings often remain elusive. We previously discovered that disrupting *Vibrio cholerae’s* ability to eat glucose (by disrupting the *pgi* gene) resulted in a damaged cell envelope and enabled us to kill *V. cholerae* more easily using antibiotics like penicillin. Upon deletion of *pgi*, glucose-phosphate levels rapidly build up and inhibit the enzymatic activity of GlmU, a key step of bacterial peptidoglycan precursor synthesis. GlmU inhibition causes enhanced killing by antibiotics and a pronounced cell envelope defect. Thus, GlmU serves as a prime target for novel drug development. This research opens new routes through which central metabolism and sugar-phosphate toxicity modulate antibiotic susceptibility.

## INTRODUCTION

The antibiotic susceptibility spectrum, including resistance, tolerance, and persistence, continues to be a massive clinical threat (1, 2). Ranging from outright resistance, that is, growth in the presence of antibiotics, to bacterial “languishing” (surviving and tolerating exposure to antibiotics for a prolonged time), to having only a subset of the bacterial population persist and able to survive antibiotic exposure, there are many ways in which bacteria can respond to antibiotics (3–5). Importantly, these pathways can be consequentially related, as tolerance and persistence are stepping stones toward full resistance (6, 7). Discovering and understanding these midway points is needed to find novel potential antibiotic development routes in order to solve this pressing issue facing our health care system.

With many common antibiotic development routes already being explored with varying degrees of success, interest in processes long overlooked (like tolerance) has recently surged. Particularly, the contribution of central metabolic pathways to antibiotic susceptibility and infection outcomes has received considerable attention (8–13). Central carbon metabolism, for example, consists of interconnected pathways that ultimately produce energy for life as well as crucial precursors for biosynthesis of key macromolecules; most of these pathways are almost universally conserved (14–16). Glycolysis (more specifically, the Embden-Meyerhof-Parnas [EMP] pathway), the TCA cycle, and the pentose phosphate pathway are all vital carbon utilization networks found in virtually all extant species (17). Their interaction with many other cellular pathways (including cell envelope homeostasis) makes central carbon metabolism a potential central hub for determining antibiotic susceptibility, and consequently for developing novel forms of therapeutic intervention.

In a previous study, we discovered that deletion of *pgi*, a key enzyme in central carbon metabolism (EMP pathway) involved in the bidirectional conversion of glucose-6P (G6P) to fructose-6P (F6P), causes cell wall damage and an increase in susceptibility to cell wall-acting antibiotics in the hypertolerant gram-negative pathogen *Vibrio cholerae* (10). We found that these defects were associated with the intracellular accumulation of sugar phosphates and could be relieved by the addition of the external cell wall precursor, N-acetylglucosamine (GlcNAc). Here, we sought to determine the molecular mechanism underlying sugar toxicity in the Δ*pgi* mutant. Genetic, metabolomic, and biochemical evidence suggests that glucose-1-phosphate (G1P) inhibits GlmU function, thereby compromising the formation of UDP-GlcNAc, which compromises peptidoglycan (PG) and potentially lipopolysaccharide (LPS) biosynthesis. Our data thus identify a new potential antibiotic target in *V. cholerae*, supporting the idea that metabolic disruptions could be weaponized to combat antibiotic tolerance and resistance.

## RESULTS

### Glucose toxicity in a Δ*pgi* mutant manifests as morphological and functional damage to the cell envelope

**Fig 1:**
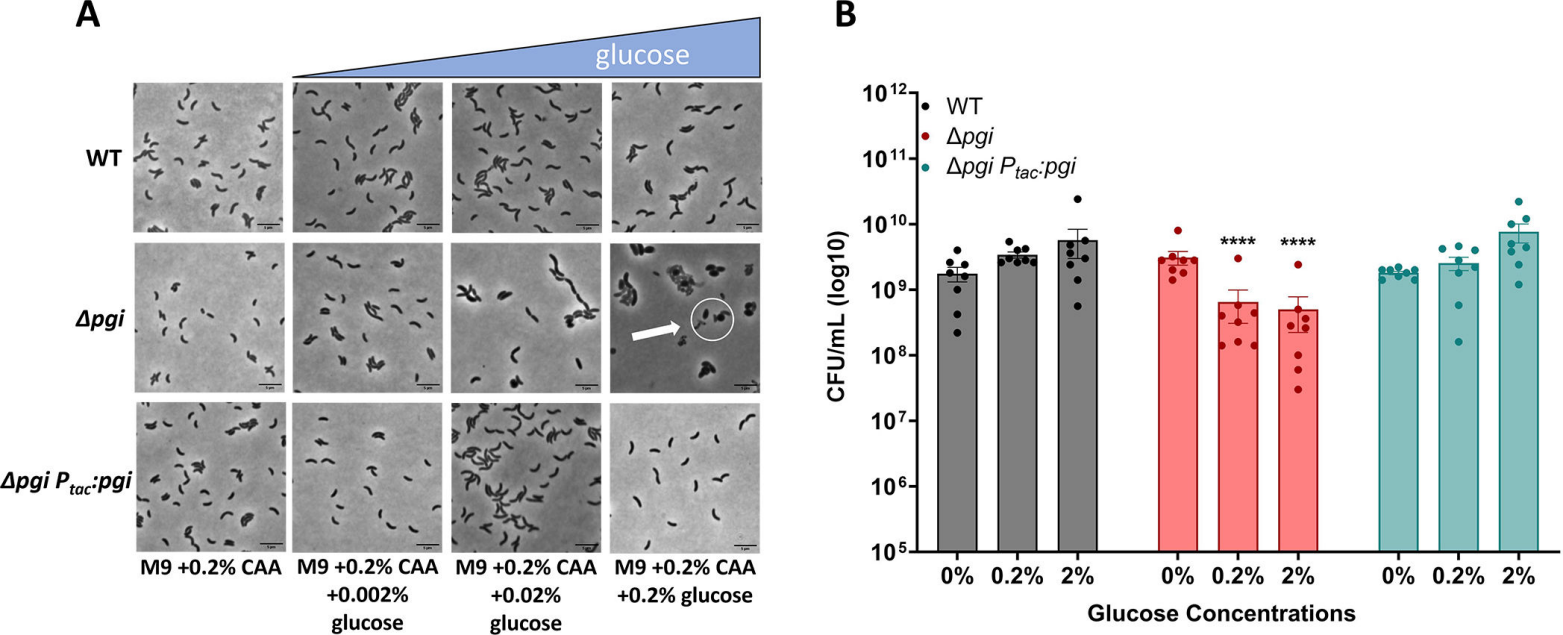
Glucose toxicity in Δ*pgi*. (**A**) Cells were imaged after 3 h of growth in the indicated conditions. Scale bar = 5 µm. White circle indicates aberrant cell morphologies. (B) CFU measurements from serially diluted cultures plated on M9 + 0.2% casamino acids agar after 3 h of exposure to the indicated glucose concentrations. Mean with SEM plotted with seven independent biological replicates. **P < 0.01, ***P < 0.001, ****P < 0.0001 (two-way ANOVA).

To understand the negative effects caused by the addition of glucose in a Δ*pgi* mutant, we observed cellular morphology and measured survival in response to increasing glucose concentrations. Wild-type (WT), ∆*pgi*, and its complemented derivative were grown to exponential phase in M9 (minimal medium) supplemented with 0.2% casamino acids, followed by the addition of increasing concentrations of glucose (0%, 0.02%, 0.2%, and 2%). Phase contrast microscopy after 3 h of growth at 37°C revealed a notable morphology defect in Δ*pgi* (Fig. 1A), in essence recapitulating our previous observations in a more defined medium (10). An increase in glucose concentrations correlated with enhanced apparent cell death (visible as cell debris) and morphological defects, a typical response to inhibition of cell wall synthesis (18–21). To probe cellular lysis further, we plated stationary phase cells on an LB agar plate with 0.2% glucose and 20 µg/mL of the cell impermeable β-galactosidase (LacZ) substrate CPRG (chlorophenol red-β-d-galactopyranoside) and incubated the plate overnight. Lysed cells will leak cytoplasmic LacZ into the medium, resulting in a deep-red color change upon CPRG processing (22). While the Δ*pgi* mutant was able to form colonies on this plate, it demonstrated markedly enhanced cell lysis in the presence of glucose (Fig. S1 at https://doi.org/10.5281/zenodo.15733387). The combination of morphological defects and lysis suggests that both LPS and PG synthesis are at least partially affected in glucose-treated *pgi* mutants, as ordinarily PG synthesis inhibition results in spheroplast formation and little lysis in *V. cholerae*.

We next sought to assess the viability of Δ*pgi* cells in increasing glucose concentrations to determine the extent of glucose toxicity. Following the same experimental setup as described previously, increasing glucose concentrations were added to the medium containing M9 + 0.2% casamino acids. The cells were then incubated for 3 h, followed by serial dilution and plating on M9 agar containing 0.2% casamino acids for CFU/mL enumeration. We noticed a slight but significant decrease in cell viability in a Δ*pgi* mutant that worsened with increasing glucose concentrations, reaching 10-fold at 2% glucose, while the WT remained unaffected (Fig. 1B). Collectively, these data indicate that glucose toxicity in a Δ*pgi* mutant results in a damaged cellular envelope.

### External N-acetylglucosamine is the sole carbon source to rescue Δ*pgi* in glucose

**Fig 2:**
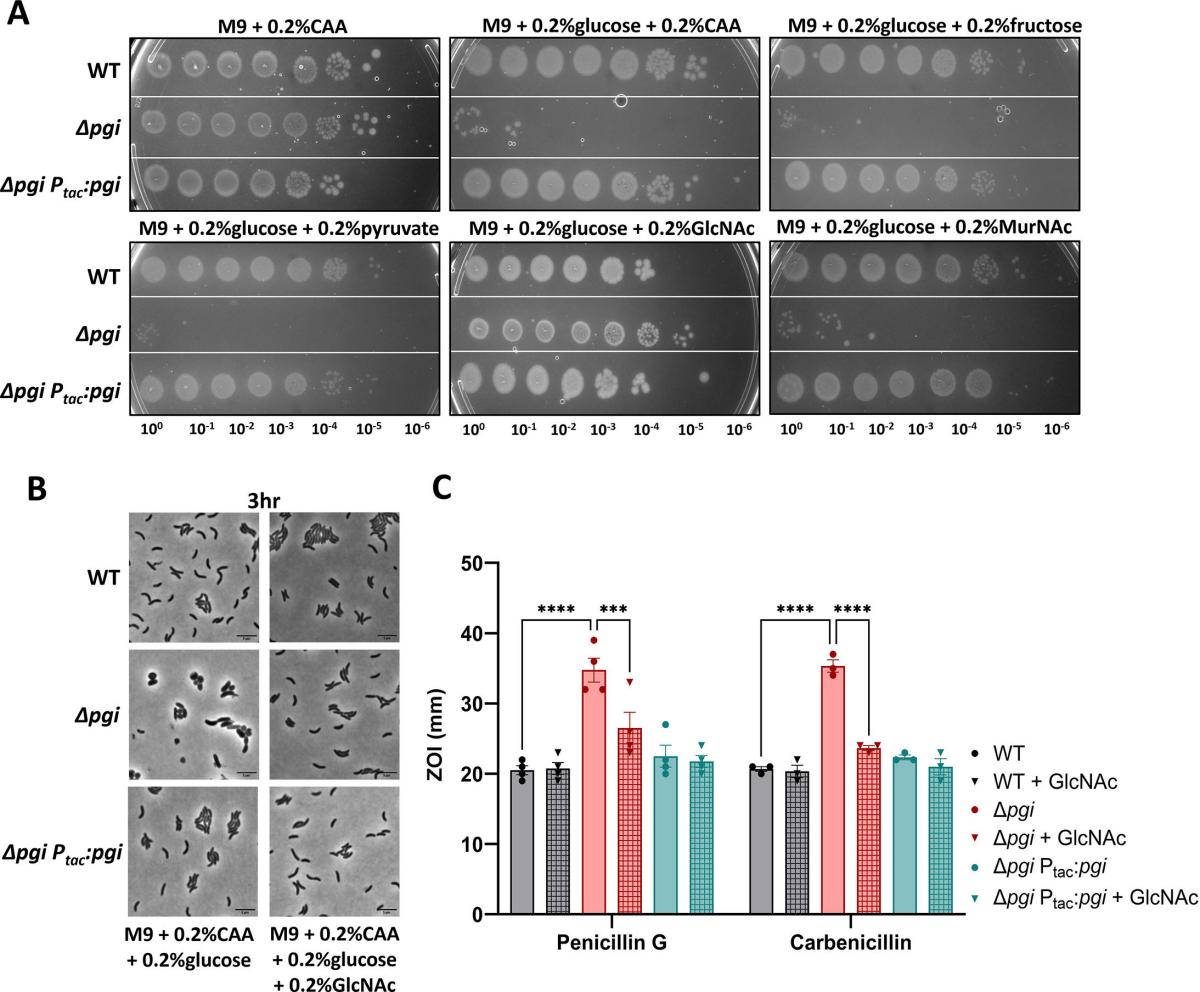
External GlcNAc is the sole carbon source to complement Δ*pgi* in glucose**.** (**A**) Serial dilutions of the indicated strains were plated on M9 minimal media supplemented with the indicated carbon sources and grown overnight at 37°C. (**B**) The indicated mutant strains were imaged after 3 h of growth at 37°C. Bar  =  5  µm. (**C**) Zone of inhibition measurements from a disk diffusion experiment on LB agar with or without the addition of 0.2% GlcNAc. Concentrations of the noted antibiotics are listed in Methods and Materials. Data represent at least three independent biological replicates; raw data points are shown with bars depicting mean with SEM. ***P < 0.001, ****P < 0.0001 (two-way ANOVA).

**Fig 3:**
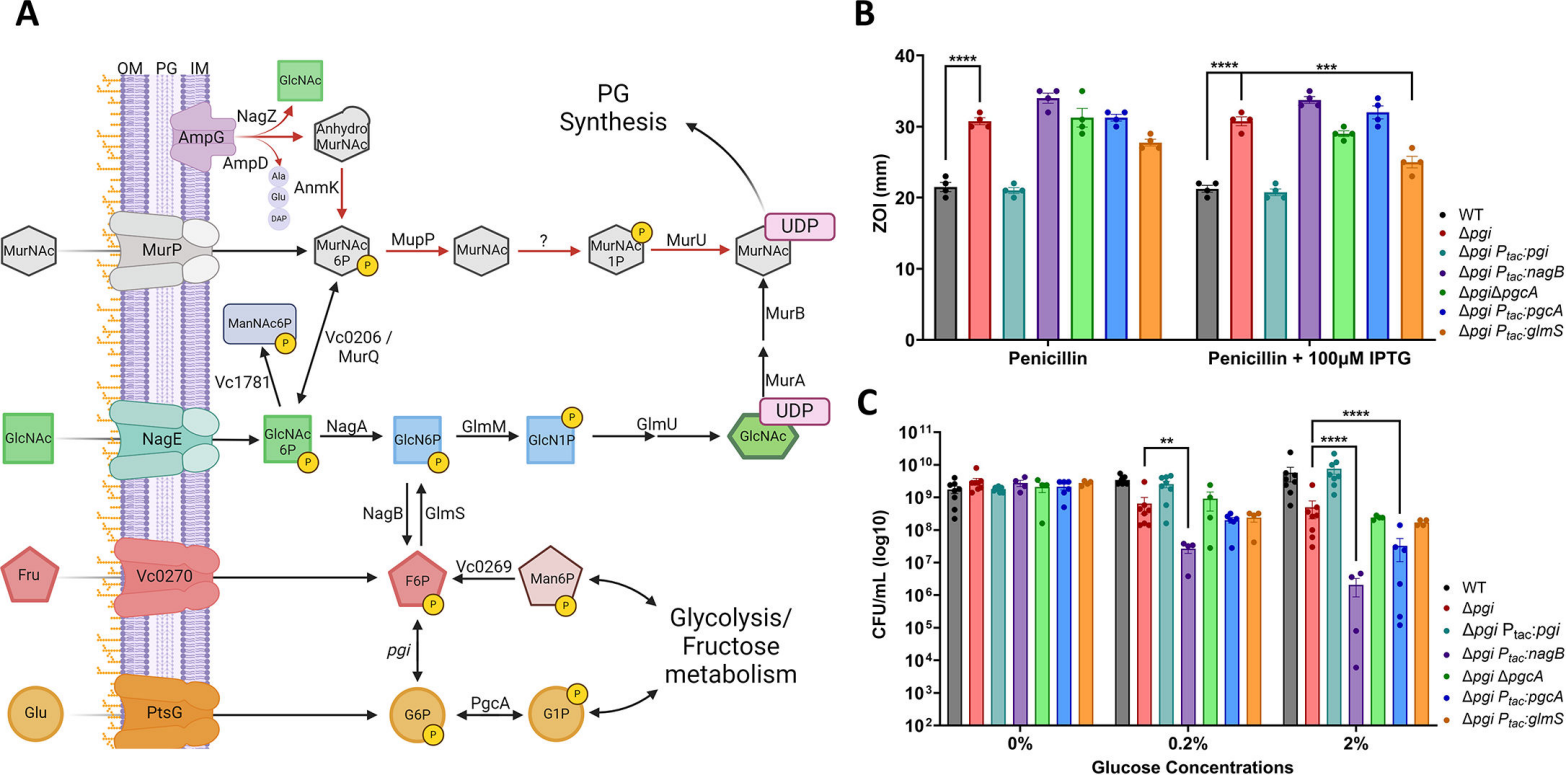
Genetic manipulation of biosynthesis pathways reveals an essentiality of glucosamine-6P in Δ*pgi*. (**A**) A metabolic pathway diagram highlighting the PG recycling pathway, the conversion of glycolytic intermediates toward PG synthesis, and GlcNAc import. Glucose (Glu) can be imported through PtsG and readily converted to Glucose-6P (G6P). PgcA can also convert glucose-1P (G1P) and glucose-6P (G6P) interchangeably. Fructose (Fru) can be imported through *vc0270*, a member of the PTS system. Pgi interconverts G6P and F6P. Mannose-6P (Man6P) can be siphoned from central metabolism toward F6P. NagB and GlmS act as a bridge away from and toward PG synthesis, respectively. GlcNAc is imported through NagE and converted to glucosamine-6P (GlcN-6P) by NagA. GlmM and GlmU build the PG precursor UDP-GlcNAc, while MurA and MurB add some additional steps to create UDP-MurNAc. MurNAc can also be imported through MurP. As MurNAc-6P, this molecule can either be funneled back toward PG synthesis directly, through MupP, MurU, and an uncharacterized enzyme, or recycled back into GlcNAc-6P by MurQ/*vc0206*. AmpG, a periplasmic PG fragment importer, can also supply internal GlcNAc from degraded cell wall products. (**B**) Zone of inhibition data from treatment with PenG with and without overexpression induction. Statistical significance was evaluated via two-way ANOVA from at least five independent replicates (raw data points shown). ***P < 0.001, ****P < 0.0001. (C) Colony formation after 3 h of glucose exposure with the indicated strains was plated on M9 + 0.2% CAA. Data for WT, Δ*pgi*, and complemented strain are reproduced from Fig. 1 for comparison. Statistical significance was evaluated via log-transformed, two-way ANOVA with four independent replicates (raw data points shown). *P < 0.1 **P < 0.01, ***P < 0.001, ****P < 0.0001.

We previously found that in LB medium (which unexpectedly contains ~10 µM glucose), the *pgi* mutant accumulates mM concentrations of glucose-6-phosphate, which causes cell envelope damage that can be rescued by adding exogenous N-acetylglucosamine (GlcNAc) (10). However, since these experiments were done in LB (which produces a messy, poorly characterized physiology [23]), we sought to utilize a more chemically defined growth medium. In principle, the observed growth defect of Δ*pgi* in glucose could be due to either a decrease in cell wall precursor synthesis (which branches from glycolysis at fructose-6-phosphate, the product Pgi generates), or reduced flux into lower glycolysis (summarized in Fig. 3A), which could diminish reducing equivalents and ATP/GTP. Indeed, glucose phosphate toxicity (albeit in response to the glucose phosphate analog alpha-MG) in *E. coli* can be overcome by adding glycolytic intermediates, including fructose-6-phosphate (24). To test these ideas, we supplemented Δ*pgi* grown on M9 + 0.2% glucose with a panel of carbon sources, covering the spectrum of glycolysis and cell wall synthesis (Fig. 2A). After confirming cells could import and utilize these different carbon sources using a growth assay (Fig. S2A at https://doi.org/10.5281/zenodo.15733387), we then plated serial dilutions of overnight cultures (grown in M9 + 0.2% casamino acids) on M9 agar plates with glucose and the respective carbon sources to assess rescue effects on the *pgi* mutant.

Interestingly, only GlcNAc could rescue Δ*pgi* growth and morphology in the presence of glucose (Fig. 2A and B). Neither fructose (which, upon import, gets converted to fructose-6-phosphate), pyruvate, nor succinate or glycerol rescued the ∆*pgi* growth defect on glucose, suggesting that ∆*pgi* glucose susceptibility is not primarily due to disruption of glycolysis. We previously reported that a Δ*pgi* mutant was more sensitive to cell-wall targeting antibiotics (10). Therefore, we also sought to investigate the effect of GlcNAc on antibiotic susceptibility. Here, we added 0.2% GlcNAc to LB agar plates and measured the zone of inhibition in response to two β-lactam antibiotics (penicillin G and carbenicillin) (Fig. 2C). Addition of GlcNAc increased resistance to the ∆*pgi* mutant (though not to the WT). Thus, GlcNAc supplementation restores all characterized defects of a Δ*pgi* mutant.

Interestingly, MurNAc, another cell wall fragment, did not restore growth (Fig. 2A). This was curious, as both external MurNAc and GlcNAc should converge in the same pathway. However, MurNAc import feeds into a pathway more upstream of *pgi* activity than GlcNAc and requires more steps to convert it into the common cell wall precursor glucosamine-6P (GlcN-6P). It is possible that the enzyme responsible for converting MurNAc-6P to GlcNAc-6P, MurQ, or the MurNAc transporter, MurP, is too inefficient for restoring optimal carbon flux, or not expressed under our growth conditions. Consistent with an inefficiency of MurNAc utilization, growth on MurNAc as the sole carbon source resulted in much poorer yield than growth on GlcNAc (Fig. S2 at https://doi.org/10.5281/zenodo.15733387).

### Genetic manipulation of biosynthesis pathways reveals PG precursor demand in Δ*pgi*

We then began to genetically explore the GlcNAc import pathway and its connections between *pgi* and cell wall synthesis (summarized in Fig. 3A). We previously reported that G6P accumulates in a *pgi* mutant; however, our untargeted metabolomics approach could not distinguish between G6P and G1P. To dissect these two sugar phosphate species further, we constructed gene deletion and overexpression strains of the enzyme that bidirectionally converts G6P into G1P, *pgcA* (vc2095), also known as pgm in *Escherichia coli,* in a Δ*pgi* background. In *Bacillus subtilis*, *pgcA* is shown to favor the conversion of G1P, and we presumed the same to be happening in *V. cholerae* (25). We hypothesized that reducing (through gene deletion) or boosting (by overexpression) glucose-phosphate levels might mitigate or exacerbate *pgi* mutant phenotypes. We were additionally interested in the branch point enzymes NagB (*vca1025*) and GlmS (*vc0487*) for their role in regulating flux between glycolysis and cell-wall synthesis. We reasoned that by siphoning away early PG precursor metabolites (i.e., GlcN-6P) into glycolysis (via NagB), a *pgi* mutant would experience more defects, while GlmS overexpression should have the opposite effect. We thus tested these strains for their ability to modulate Δ*pg*i phenotypes. First, we tested PenG antibiotic susceptibility by measuring the zone of inhibition (Fig. 3B). *glmS* overexpression significantly reduced Δ*pgi* PenG sensitivity, suggesting that directing metabolic flux toward PG precursors and away from glycolysis was beneficial. Conversely, overexpression of *nagB* and *pgcA* tendentially enhanced PenG sensitivity, though this was not statistically significant (Fig. 3B).

Next, we turned to another phenotype, that is, ∆*pgi*’s reduced survival in the presence of glucose. We thus treated the strains with either 0.2% or 2% glucose for 3 h and then plated on M9 + 0.2% casamino acids (Fig. 3C). In both glucose concentrations, overexpressing *nagB* in a Δ*pgi* background resulted in significantly lower cell viability. At higher glucose levels, overexpression of *pgcA* in Δ*pgi* also became statistically significant in reducing cell viability. However, the deletion of *pgcA* did not rescue the *pgi* mutant, perhaps suggesting an alternative route to G1P production, as observed in other bacterial species (26–28). Collectively, these data point to a contribution of G1P in sugar phosphate toxicity of ∆*pgi* and suggest that carbon flux away from glycolysis helps this mutant, while flux away from cell wall precursor synthesis exacerbates its growth defect.

### Targeted metabolomics suggests a metabolite bottleneck around GlmU in glucose-treated *pgi* mutant cells

**Fig 4:**
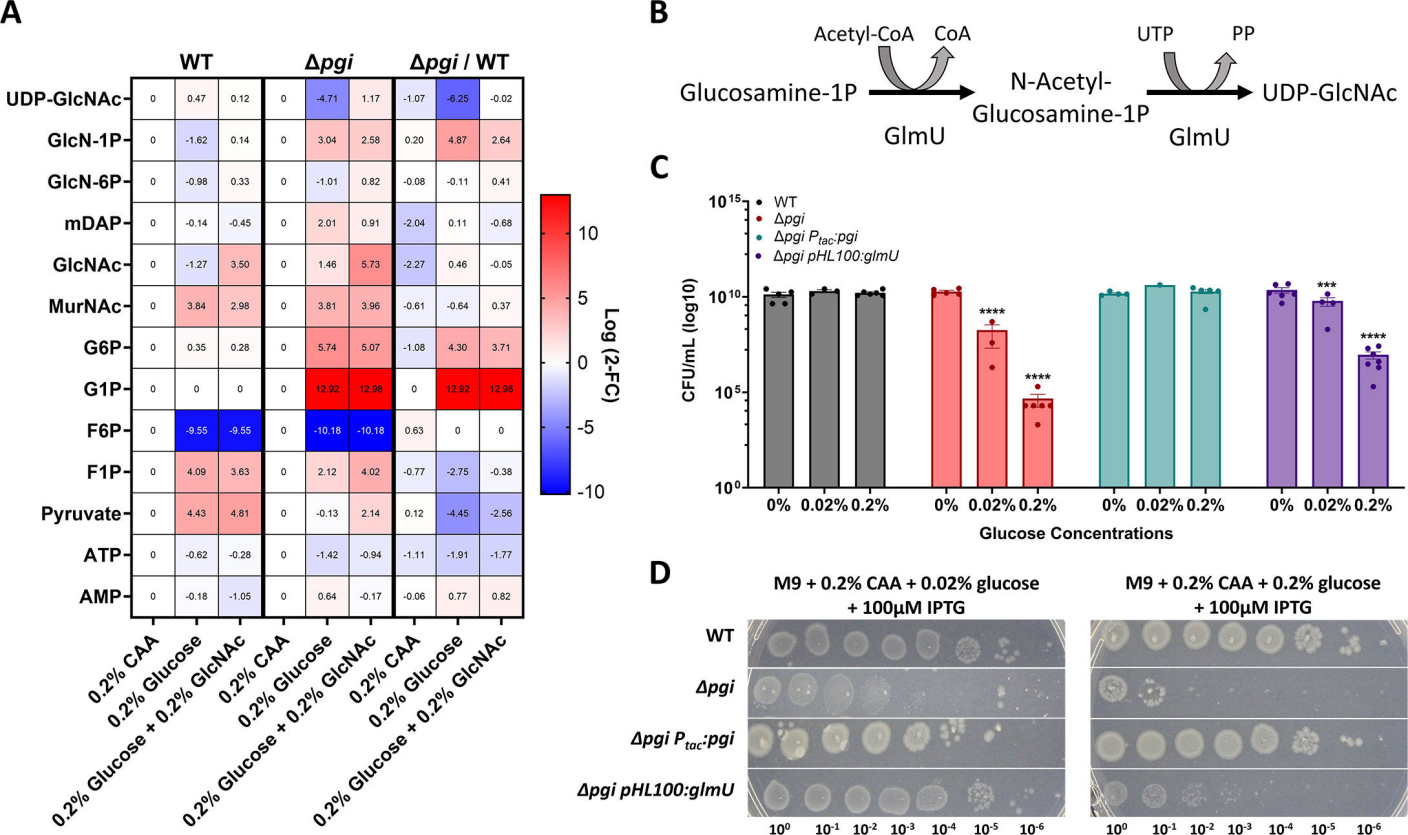
Targeted metabolomics of Δ*pgi* reveals bottleneck around GlmU activity. (**A**) Heatmaps normalized to strain-specific casamino acids conditions. The right column represents the change between strains in the indicated conditions. Log2 fold change is shown on the right side of the maps. (**B**) The enzymatic reaction of GlmU. The acetyltransferase activity catalyzes N-acetylglucosamine-1P from GlcN-1P and acetyl-CoA. The second step is the uridyltransferase reaction, which adds UDP onto GlcNAc, forming the end-product UDP-GlcNAc. (**C**) Overnight cultures were serially diluted and spot-plated on M9 agar with the indicated additions of percent glucose and 100 µM IPTG. At least four independent replicates are presented, with raw data points and SEM. ***P < 0.001, ****P < 0.0001 (two-way ANOVA). Δ*pgi* was compared against WT values and Δ*pgi* pHL100: glmU was compared against Δ*pgi*. (**D**) The indicated strains were grown overnight in M9 + casamino acids and then diluted and spot-plated on M9 agar plates containing CAA and the indicated glucose concentration.

To further characterize the metabolic disruptions observed in a Δ*pgi* mutant, we conducted targeted metabolomics upon glucose exposure in M9 medium + CAA. Three hours after addition of either 0.2% glucose or a combination of 0.2% glucose and 0.2% GlcNAc, cells were pelleted, and metabolites were extracted using methanol. Samples were analyzed using LC-MS (see Materials and Methods), and peaks were compared to pure chemical standards. Upon normalizing the data to the casamino acid conditions, we noted a sharp increase in G1P and G6P in the mutant, consistent with the *pgi* mutant’s inability to metabolize glucose through the EMP glycolysis pathway. The WT, but not ∆*pgi*, experienced an increase in pyruvate levels upon glucose addition. The combination of increased pyruvate and reduced G6P/G1P levels (and indeed very low F6P levels) in the WT perhaps indicates highly efficient upper glycolysis, which encounters a bottleneck at pyruvate processing. The relative lack of increase in pyruvate upon glucose addition in ∆*pgi* suggests that non-EMP pathways for glucose utilization (e.g., pentose phosphate pathway) may be inefficient in *V. cholerae,* at least under the conditions tested here. We also observed a sharp rise in GlcN-1P levels (16-fold change), and a sharp, 36-fold decrease in UDP-GlcNAc, when Δ*pgi* was grown in medium containing glucose, compared to WT (Fig. 4A). Thus, the described sugar toxicity likely results from the inhibition of the enzyme converting GlcN-1P to UDP-GlcNAc, which is GlmU (Fig. 4B). This bottleneck was slightly improved upon the addition of GlcNAc to the growth medium, suggesting GlcNAc-mediated relief of this inhibition. As expected from the pathway metabolizing external GlcNAc (Fig. 3A), the addition correlated with a slight increase in GlcN-6P (into which external GlcNAc is converted by *V. cholerae* through the action of the NagE transporter and NagA deacetylase) (29).

Plausibly, the accumulation of G1P and/or G6P in the Δ*pgi* mutant competitively inhibits GlmU, downregulating cell wall synthesis. There is evidence of a similar effect in *Mycobacterium tuberculosis*, where at least G1P competitively inhibits GlmU *in vitro* (30). G1P levels did not change (Fig. 4A) upon addition of GlcNAc, which may indicate that GlcNAc addition does not stop sugar phosphate accumulation, but rather circumvents PG precursor synthesis inhibition by supplying more substrate (which would suggest competitive inhibition). Fructose-6P levels in both WT and Δ*pgi* cells were significantly lower in glucose-containing medium. It is possible that flux into the TCA cycle and downstream respiration is faster in the presence of glucose, which causes a net relative decrease in F6P levels relative to growth in casamino acids (gluconeogenic conditions).

If GlmU is competitively inhibited by glucose phosphates, it should be possible to circumvent this inhibition by increasing the abundance of GlmU. Therefore, we next sought to genetically explore the role of GlmU by creating an overexpression construct in a Δ*pgi* background. Overexpression of *glmU* from a high copy number plasmid (see Materials and Methods) caused a significant increase in cell viability in the presence of glucose in comparison to Δ*pgi* alone (Fig. 4C). While there still was a decrease in overall survival, even when expressing *glmU* excessively, this can be explained by the glucose phosphate inhibition logic. We previously measured an over 200× increase in G1P/G6P in Δ*pgi* when grown in LB (10); adding a few more GlmU molecules is likely not sufficient to compensate for the intracellular flooding of G1P/G6P, especially when grown in medium with high glucose concentrations. We also overexpressed *glmU* from an overnight culture and plated serial dilutions on M9 + 0.2% CAA supplemented with either 0.02% glucose or 0.2% glucose (Fig. 4D). GlmU overexpression significantly improved ∆*pgi* plating efficiency on glucose, particularly at the lower glucose concentration, again supporting the idea of GlmU being the target of glucose toxicity in the ∆*pgi* mutant.

### *In vitro* biochemical validation of GlmU inhibition by G1P

**Fig 5:**
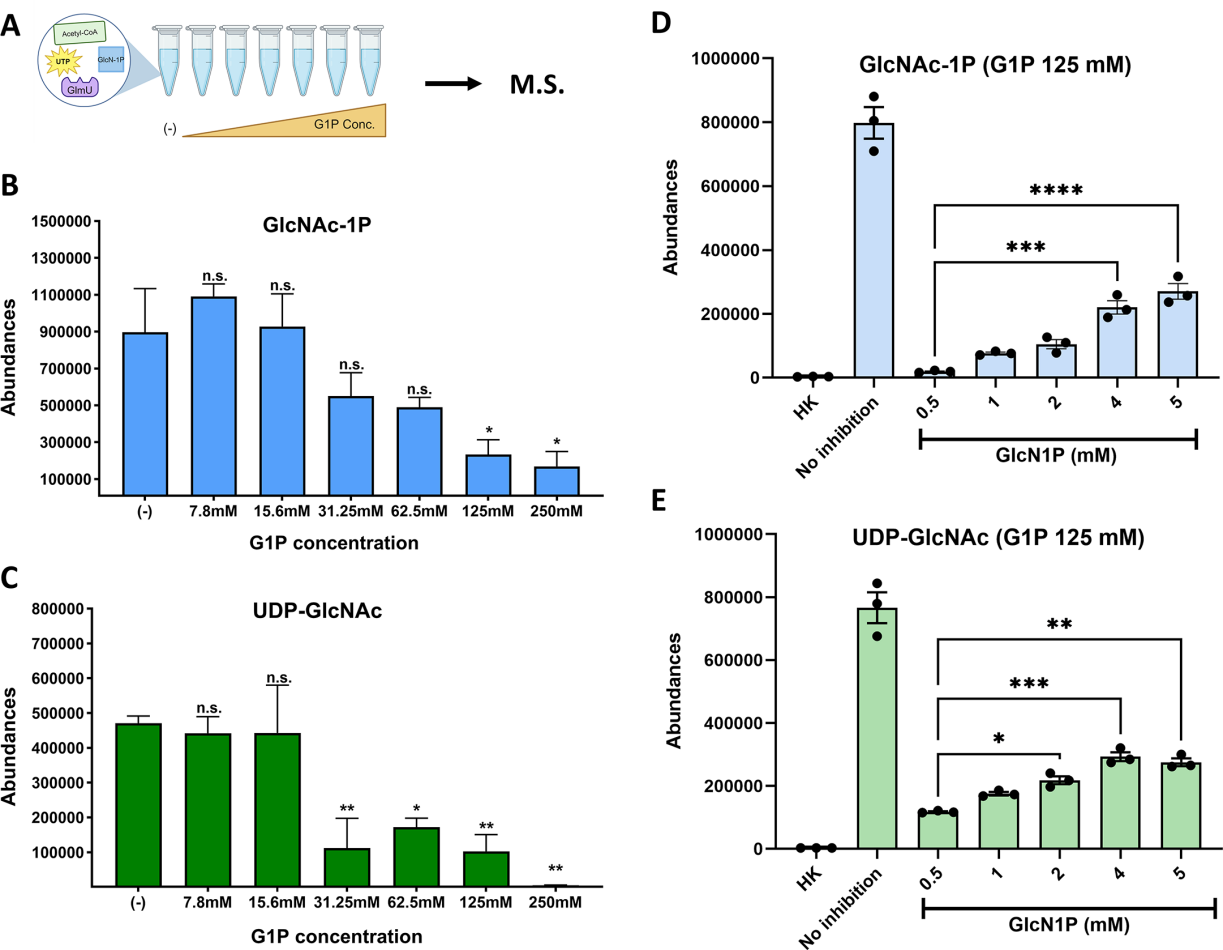
Glucose-1P competitively inhibits GlmU biochemical reaction in a concentration-dependent manner. (**A**) Schematic portraying the *in vitro* biochemistry experimental design. Biochemical reaction components, UTP, acetyl-CoA, GlcN-1P, and GlmU, mixed with buffer, were added to test tubes. After incubation, the reaction was stopped and ran on the LC-MS machine. Abundance peaks were measured for the stated molecules. (**B**) Peak values for GlcNAc-1P product from reactions with increasing glucose-1P concentrations. SEM plotted with three replicates. (**C**) Peak values for UDP-GlcNAc product from reactions with increasing glucose-1P concentrations (in mM, *X*-axis). Averages and SEM plotted from three replicates. (**D**) Peak values for GlcNAc-1P from reactions with increasing GlcN-1P addition and constant G1P addition at 125 mM. SEM plotted with three replicates. (**E**) Peak values for UDP-GlcNAc from reactions with increasing GlcN-1P concentrations and constant G1P addition at 125 mM. SEM plotted with three replicates. *P < 0.05, **P < 0.01, ***P < 0.0005 , ****P < 0.0001 (one-way ANOVA with multiple comparisons).

We next conducted an *in vitro* biochemistry assay using purified *V. cholerae* GlmU to test whether G1P/G6P inhibition was direct. To this end, we tested the inhibitory activity of G1P and G6P in a biochemical assay containing purified GlmU, as well as the reactants acetyl-CoA, GlcN-1P, and UTP (Fig. 5A). G1P and G6P concentrations were designed to mimic the glucose concentrations previously measured in Δ*pgi* (10). After 30 min at 30°C, the reaction was stopped and analyzed for the emergence of the product of the GlmU reaction, UDP-GlcNAc, using LC-MS. Upon the addition of increasing concentrations of G1P, but not G6P (Fig. S3A at https://doi.org/10.5281/zenodo.15733387) to the reaction mix, we observed a significant decrease in UDP-GlcNAc abundance starting at 31.25 mM of G1P, and near-complete inhibition at 250 mM (Fig. 5C). We also measured the intermediate product of GlmU’s bifunctional reaction, GlcNAc-1P (Fig. 5B). We found that at higher concentrations of G1P, GlcNAc-1P was also significantly reduced, but not eliminated. The lack of accumulation of this intermediate product suggests that acetyltransferase activity (the first reaction) is inhibited rather than the uridyl-transferase activity (the second reaction). The stronger inhibitory effect of G1P on the final product UDP-GlcNAc is also consistent with this, as the reduced quantity of the intermediate GlcNAc-1P is potentially not enough to saturate the active site of the downstream reaction. This would explain why a higher dosage of G1P (250 mM) is needed to have a significant reduction in GlcNAc-1P, while only 32.5 mM of G1P is needed to inhibit UDP-GlcNAc production.

Next, we predicted that if G1P inhibits GlmU competitively, we should be able to relieve this inhibition by increasing the substrate concentration. We thus repeated our biochemical assay in the presence of 125 mM G1P (full inhibition) and then added increasing concentrations of GlcN-1P. We then quantified the products GlcNAc-1P and UDP-GlcNAc. A 10-fold increase in substrate concentration above baseline yielded a robust and statistically significant 10-fold increase in the intermediate product (GlcNAc-1P) and 2-fold increase in UDP-GlcNAc (Fig. 5D and E). We also confirmed the lack of inhibition with G6P in the same assay (Fig. S3B and C at https://doi.org/10.5281/zenodo.15733387). Thus, it is highly likely that G1P competitively inhibits GlmU, which can be rescued via G1P replacement with natural substrate.

### Molecular modeling reveals putative target site for glucose phosphate inhibition

**Fig 6:**
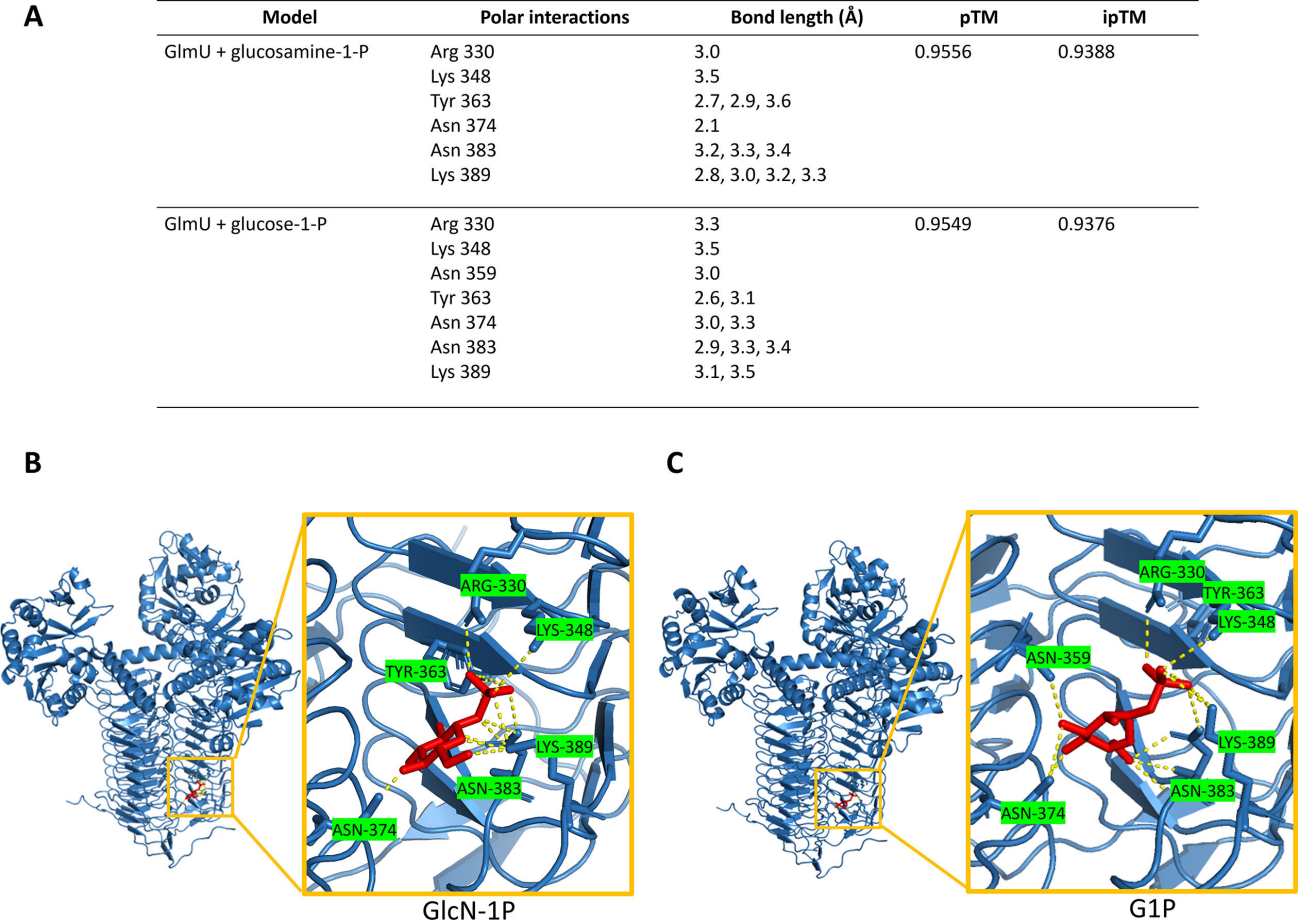
Molecular modeling reveals putative target site for glucose phosphate inhibition. (**A**) Residues predicted to interact with glucosamine-1-P and glucose-1-P, predicted template modeling (pTM) and interface predicted template modeling (ipTM). (**B**) Molecular modeling of GlmU binding glucosamine-1-P (red) and the associated polar interactions. (**C**) Molecular modeling of GlmU binding glucose-1-P (red) and the associated polar interactions.

We next sought to model the observed inhibition of G1P against GlmU. To this end, we turned to Chai Discovery, which can predict both protein multimers and protein-ligand binding (31). We obtained multiple models of predicted binding sites for both GlmU’s natural substrate, GlcN-1P, and also its presumed inhibitor, G1P, within the trimeric form of GlmU. Visualization of these molecular models revealed that GlcN-1P engages in polar interactions with Arg330, Lys348, Tyr363, Asn374, Asn383, and Lys389 (Fig. 6A–C). This model returned pTM and ipTM scores of 0.9556 and 0.9388, respectively; pTM scores > 0.5 and ipTM scores > 0.8 are considered confident predictions (32). Sequence alignments with GlmUEC^EC^ and GlmUMtb^Mtb^, which have well-characterized active sites (30, 33), indicate that the modeled residues likely form the acetyltransferase active site in GlmUVC^VC^ (Fig. S4A–C at https://doi.org/10.5281/zenodo.15733387). Additionally, structural alignments of GlmUVC^VC^ to GlmUEC^EC^ and GlmUMtb^Mtb^ indicate that the structure of GlmU is highly conserved across diverse organisms. Molecular modeling of the putative interaction between GlmU and G1P revealed a binding site at the same location as the natural substrate, indicating that G1P may competitively inhibit GlmU (Fig. 6A). This model returned similarly high pTM and ipTM scores (0.9549 and 0.9376, respectively). While there were some predictions that modeled G1P within the uridyltransferase pocket, the strength of the polar interactions was not as robust as the models that predicted G1P to bind within the acetyltransferase pocket (Fig. S5B at https://doi.org/10.5281/zenodo.15733387). We additionally examined the predicted binding with G6P and found while it is predicted to bind in a similar fashion as G1P, the pTM and ipTM scores were lower (Fig. S5C and D at https://doi.org/10.5281/zenodo.15733387). How the phosphate location on the glucose molecule elicits such a drastically different inhibition response remains to be explored. Taken together, this suggests that G1P accumulation competitively inhibits the acetyltransferase activity of GlmU.

## DISCUSSION

Sugar-phosphate stress and its associated cellular defects remain underexplored in the context of antibiotic susceptibility. Here, we present data that help elucidate the mechanism of glucose poisoning in *V. cholerae*. We previously found that a *pgi* mutation results in pronounced cell wall damage and concomitant increase in susceptibility to β-lactam antibiotics (10). In this study, we show that glucose toxicity in ∆*pgi* is due to (likely competitive) inhibition of GlmU (a key step in PG precursor synthesis) by sugar phosphate species. Sugar-phosphate toxicity has been studied for the past seven decades (34–39), yet the mechanisms for glucose-related toxicity appear to be diverse, species-dependent, and poorly understood (40, 41). In *B. subtilis,* a mutant defective in both glycolysis and pentose phosphate pathway builds up excessive G1P, which was suggested to inhibit an early PG precursor step, resulting in cell lysis (37). However, this observation was never followed up mechanistically. *E. coli* strains with a defective *pgi* experience significant sugar-phosphate stress, resulting in post-transcriptional regulation of *ptsG* to reduce sugar intake (41). While G6P levels are elevated in Δ*pgi* backgrounds in *E. coli*, there is no observable cell-wall damage, and toxicity appears to be due to diversion of resources from glycolysis, rewiring of metabolism, and possibly redox imbalance stress (38, 39, 42–44). The reduced glucose phosphate toxicity in *E. coli* may be due to a more enhanced flux into the pentose-phosphate pathway in this species, which could in principle efficiently remove G6P (44). Additionally, *E. coli* is known to have a robust glucose-phosphate stress response system regulated by small RNA molecule SgrS and its activator protein, SgrR (24, 45, 46). SgrS controls import of glucose through the PTS system by modulating *ptsG*. While *V. cholerae* encodes an SgrR homolog, the small RNA SgrS has not been identified.

Our data clearly show that sugar phosphate toxicity can directly contribute to cell envelope defects. Our combined genetic, biochemical, and modeling data point to competitive inhibition of GlmU by G1P, which could be explained by the similarity in structure between G1P and GlmU’s natural substrate GlcN-1P (Fig. 6). Additionally, if G1P is targeting the acetyltransferase domain, the alleviating effect of GlcNAc makes sense, as the external GlcNAc would readily be converted to first GlcN-6P and then GlcN-1P. More GlcN-1P would outcompete the G1P and restore GlmU functionality.

These findings more broadly shed light on the importance of central metabolism as a potential source of novel antibiotic targets. GlmU is a well-conserved protein among highly relevant pathogens, most notably *M. tuberculosis*. There have been extensive studies exploring the potential for GlmU as an anti-TB drug target, but little has been explored in other bacteria. Some studies have identified high-throughput methods and computational models for drug screening against GlmU (47–52), while others have investigated the effect of depleting GlmU in infection models, mimicking the potential effects an inhibitor might have (53–58). In principle, UDP-GlcNAc biosynthesis serves as an ideal drug target, as it is required for not only PG, but also LPS biosynthesis. By designing targets for novel antibiotics that disrupt more than one biochemical pathway, resistance and mutations leading to reduced efficiency are less likely to occur.

## MATERIALS AND METHODS

### Bacterial strains and growth conditions

All *V. cholerae* strains used in this study are derivatives of *V. cholerae* El Tor strain N16961 and are summarized in Table S1 (https://doi.org/10.5281/zenodo.15733387). *V. cholerae* was grown on Luria-Bertani (lysogeny broth) (LB) medium (for a 1 L bottle, 10 g Casein peptone, 5 g yeast extract, 10 g NaCl, and 12 g agar, all from Fischer Bioreagents) at 30°C or in M9 minimal medium (for a 1 L bottle, 15 g agar, 200 mL 5× M9 salts [for a 1 L bottle, 35 g Na_2_HPO_4_·7H_2_O, 15 g KH_2_PO_4_ , 2.5 g NaCl, 5 g NH_4_Cl], 0.5 mL 1 M MgSO_4_, 0.1 mL 1 M CaCl_2_, and 1 mL FeCl_3_/citric acid) at 37°C; 200 µg/mL of streptomycin was also added (N16961 is streptomycin resistant). Where applicable, growth media were supplemented with 0.2% glucose (wt/vol), 0.2% casamino acids (wt/vol), or 0.2% GlcNAc (wt/vol). All other carbon sources were also 0.2% (wt/vol).

For growth dynamic experiments, overnight cultures were diluted 100-fold into 1 mL growth media + streptomycin. 200 µL of this seed stock was added to wells in a 100-well honeycomb and incubated in a Bioscreen growth plate reader (Growth Curves America) at 37°C with random shaking at maximum amplitude, and OD_600_ recorded at 10-min intervals.

### Plasmid and strain construction

Plasmids and oligonucleotides used in this study are summarized in Table S2 (https://doi.org/10.5281/zenodo.15733387). *E. coli* MFDλpir (a diaminopimelic acid [DAP] auxotroph) or SM10 λpir was used for conjugation into *V. cholerae*, for gene deletions and overexpression plasmids, respectively (59). Overexpression strains were created using the chromosomal integration plasmid pTD101, a derivative of pJL1 containing lacIq and a multiple-cloning site under the control of the isopropyl-β-d-thiogalactopyranoside (IPTG)-inducible Ptac promoter, or pHL100mob, a non-integrative high copy number plasmid also inducible through IPTG (60). pTD101 integrates into the native *V. cholerae* lacZ (*vc2338*) locus. Genes for complementation experiments were amplified from N16961 genomic DNA (61), introducing a strong consensus ribosome-binding site (RBS) (AGGAGA), and cloned using Gibson assembly. Plasmids were colony PCR verified using primers 1 and 2 (pTD101) or 5 and 6 (pHL100mob). Gene deletions were constructed using the pTOX5 cmR/msqR allelic exchange system (62). In short, 500 bp regions flanking the gene to be deleted were amplified from N16961 genomic DNA by PCR and cloned into the suicide vector using Gibson assembly (63). Plasmids were colony PCR verified using primers 3 and 4. All plasmids were verified by Sanger sequence before conjugation.

Conjugation into *V. cholerae* was performed by mixing overnight cultures 1:1 (100 µL donor plus 100 µL recipient) in 800 µL fresh LB, followed by pelleting (7,000 rpm, 2 min) and resuspending in 100 µL LB. The mixture was then spotted onto LB agar (with 600 µM DAP for *E. coli* MFDλpir growth) and incubated for 4  h (overnight for pTOX5 deletions) at 37°C. Selection for single-crossover mutants was then achieved by streaking the mating mixture on either streptomycin (200 µg/mL) plus carbenicillin (100 µg/mL) (pTD101), streptomycin (200 µg/mL) plus kanamycin (50 µg/mL) (pHL100mob), or streptomycin (200 µg/mL) plus chloramphenicol (100 µg/mL), and 600 µM DAP for pTOX5 and incubating overnight at 37°C.

For pTD101 insertion, carbenicillin-resistant mutants were counterselected on salt-free LB supplemented with 10% sucrose and X-Gal (5-bromo-4-chloro-3-indolyl-β-d-galactopyranoside) (120 µg/mL) and grown at ambient temperature for 2 days. White colonies (indicating a disrupted lacZ) were isolated and PCR-verified using primers 23 and 24. For pTox-mediated recombination, chloramphenicol-resistant colonies were counter-selected on M9 minimal medium containing 2% (vol/vol) rhamnose at 30°C for 18 h. Deletions were verified by PCR using flanking and internal primers and verified with whole-genome sequencing.

### Cell viability assay and glucose time-dependent killing assay

To test cell viability, overnight cultures were added to sterile 1× PBS for serial dilution from 1:10 to 1:10^7^. Overnight cultures and diluted cultures (5 µL) were spotted for CFU/mL on different media plates, as described in the figure legend. Dried plates were then incubated at 37°C (M9 agar) overnight and counted the next day. For glucose concentration-dependent experiments, strains were grown overnight in M9 + 0.2% casamino acids at 37°C. The following day, the cultures were diluted 1:1,000 into fresh M9 + 0.2% casamino acids and incubated at 37°C for 3 h. Then, various concentrations of glucose were added to the media and incubated for another 3 h at 37°C, then serially diluted onto M9 agar + 0.2% casamino acids and left overnight at 37°C. CFU/mL were counted the next day. For microscopy, strains were grown as previously described, then imaged without fixation on M9 + 0.8% agarose pads using a Leica DMi8 inverted microscope.

### Antibiotic sensitivity assay

For zone of inhibition assays, a lawn of overnight cultures (100 µL) was spread on an LB agar plate with or without 0.2% GlcNAc and allowed to dry for 15 min. 10 µL of antibiotic solutions (100 mg/mL PenG or 100 mg/mL carbenicillin) was placed on Thermo Scientific Oxoid Antimicrobial Susceptibility Test filter disks (6 mm, product code: 10609174) onto the agar surface and incubated at 30°C overnight before measurements.

### Metabolomics

Three biological replicates were grown in M9 + 0.2% casamino acids overnight at 37°C. 1 mL of culture was pelleted (2 min, 7,000 rpm) and washed with M9 media. The cultures were then added 1:50 into 5 mL of M9 + 0.2% casamino acids and incubated at 37°C for 3 h. Following incubation, either 0.2% glucose or 0.2% glucose and 0.2% GlcNAc were added to the tubes and incubated for an additional hour at 37°C. After treatment, 2 mL of sample was taken per condition and pelleted at 7,000 rpm for 2 min. The supernatant was removed, and the cell material pellet was flash-frozen with liquid nitrogen. 200 µL of cold 80% methanol was then added to the pellets. Pellets were stored at −80°C. These pellets were lysed, and 3 µL samples were analyzed using Agilent InfinityLab Poroshell 120 HILIC-Z (Agilent 683775-924). The chromatographic separation employed two solvent phases: solvent A (water + 10 mM NH_4_OAc + 5 mM InfinityLab Deactivator Additive, pH 9, adjusted with NH_4_OH) and solvent B (85% ACN + 10 mM NH_4_OAc + 5 mM InfinityLab Deactivator Additive, pH 9, adjusted with NH_4_OH). The gradient program consisted of 0–2 min (96% B), 5.5–8.5 min (88% B), 9–14 min (86% B), 17 min (82% B), 23–24 min (65% B), 24.5–26 min (96% B), and a 10-min end-run at 96% B. Mass spectrometry was performed using an Agilent 6230 Time of Flight (TOF) mass spectrometer (MS) with an Agilent Jet Stream electrospray ionization (ESI) source in negative mode. Data analysis involved peak visualization and confirmation using Profinder 8.0 (Agilent) software and a pathway-specific, manually curated database. Standard metabolites were included in each run for retention time matching and verification. Heatmaps were generated using Prism, with averaged peak heights normalized to the control casamino acids condition.

### Protein purification

*V. cholerae’s* GlmU gene was amplified from N16961 gDNA and cloned into pET28a downstream of 6×His-SUMO tag (64). Plasmids were verified by Sanger sequencing. *E. coli* BL21 (DE3) (Novagen) was transformed with the resulting recombinant plasmid (pET28a-GlmU). Overnight cultures (10 mL) were used to inoculate 1 L of LB with kanamycin (50 µg/mL) and incubated at 37°C with vigorous shaking (220 rpm) until they reached an OD_600_ between 0.6 and 0.8. Cultures were induced with 1 mM IPTG at 18°C and 180 rpm overnight. Harvested cells were pelleted and resuspended in 15 mL of cold purification buffer (20 mM Tris, pH 7.5, 150 mM NaCl), and lysed by sonication. Lysates were cleared by centrifugation at 31,000 × *g* for 40 min at 4°C, and loaded onto a HisPur cobalt column (Thermo Scientific; Catalog no. 89964) and washed multiple times with purification buffer until protein was undetectable in the flowthrough by the Bradford reagent. The bead slurry was then transferred to a 5 mL microtube with 60 µL of ULP1 Sumo protease and digested overnight at 4°C rotating. Protein was eluted the next day with 20 mL of purification buffer. Samples were analyzed by SDS-PAGE with Coomassie blue stain and then concentrated with a 30 kD Amicon concentrator (Millipore) to 5 mL. Concentrated samples were then measured using Nanodrop.

### *In vitro* GlmU biochemistry

Reaction design was taken from reference 53. In summary, GlmU reaction substrates included GlcN-1P (5 mM), UTP (5 mM), and acetyl-CoA (5 mM). Substrates were added to a 1.5 mL Eppendorf tube with 5 µL of 10× reaction buffer (50 mM Tris-HCl, pH 7.5, 5 and 5 mM MgCl_2_). A dilution series of G1P inhibitors was added (0–250 mM) and then purified GlmUVC^VC^ was added at 2 µM, for a total volume of 50 µL. The tubes were incubated at 30°C for 30 min. Equal volumes of 40:40:20 (Acn: MeOH:H_2_O) solution were added to stop the biochemical reaction. The tubes were then centrifuged for 10 min at 15,000 rpm. Half of the volume was added to a new tube and mixed with equal volumes of LC-MS solution B. These tubes were centrifuged at 4°C, 8 min, at 15,000 rpm. 25 µL of supernatant was added to the LC-MS autosampler vials and 2 µL sample volume was resolved on a Diamond Hydride Column using a 1260 Infinity II high-performance liquid chromatography (LC) system (Agilent) coupled with an Agilent Accurate-Mass 6230 TOF-MS operating in negative mode. Two liquid phases—(i) solvent A (H_2_O + 0.2% formic acid) and (ii) solvent B (acetonitrile + 0.2% formic acid)—were used at 0.4 mL/min with the following gradients: 85% B, 0–2 min; 80% B, 3–5 min; 75% B, 6–7 min; 70% B, 8–9 min; 50% B, 10–11 min; 20% B, 11–14 min; and 5% B, 14–24 min and 10 min of 85% B for the re-equilibration. Results were collected on Agilent 6230 TOF-MS with ESI source. Profinder 8.0 (Agilent) was used for the peak abundance measurement. Final metabolites were verified by comparing retention times and mass-to-charge (*m/z*) ratios with respective standards for each substrate and product. Absolute and relative counts were calculated and plotted on GraphPad Prism software. The same experimental setup was conducted for the competitive inhibition tests. The only changes occurred in a constant addition of G1P (or G6P) at 125 mM and GlcN-1P substrate levels were supplemented from 0.5 to 5 mM.

### Molecular modeling

Interactions between GlmU, glucosamine-1P, and glucose-1P were modeled using Chai Discovery (https://www.chaidiscovery.com/blog/introducing-chai-1). We input the amino acid sequence code for VCH GlmU, from UniProt (Q9KNH7) as the protein input 3×. We then uploaded the SMILES for either glucosamine-1P or glucose-1P (PubChem). Confidence scores (pTM and ipTM) were automatically generated during this analysis. Each resulting model was visualized using PyMol. Polar interactions to adjacent amino acids were identified and measured in PyMol. Pairwise structural alignments were performed in PyMol, and RMSD values were automatically generated during this analysis. Sequence alignments and analysis were performed using UniProt (https://www.uniprot.org/align).
